# Thank you to *The Lancet Regional Health* – *Southeast Asia*'s clinical and statistical peer reviewers in 2025

**DOI:** 10.1016/j.lansea.2026.100740

**Published:** 2026-02-19

Ebrahim Abbasi

Hashem Abu Serhan

Ishag F Adam

Alayne Mary Adams

O Yaw Addo

Benojir Ahammed

Alia Ahmad

Shoaib Ahmad

Syed Masud Ahmed

Kenni Ajele

Zubair Akhtar

Nasrin Akter

Muhammad Shah Alam

Muntasir Alam

Ahmed Alsuwaidi

Claudia Alves

Yaser Mohammed Al-Worafi

Abdulmohsen Al-Zalabani

Adidja Amani

Ali Amid

Anandakumar Amutha

Benjamin Anderson

Saeed Anwar

Huluvadi Shivalingaiah Anwith

S M Yasir Arafat

Gururaj Arakeri

Raphael Araujo

Ramandeep Arora

Faheem Arshad

Ni Aryastami

Saima Asghar

Zein Assad

Ashenafi Assefa

Sachin Atre

Michael Auerbach

Charlotte Avanzi

Shally Awasthi

Sumera Aziz

Syed Khurram Azmat

Yeji Baek

Soumita Bagchi

Vicky Baillie

Jitin Bajaj

Neha Bajwa

Ahad Bakhtiari

Ioannis Bakolis

Anukumar Balakrishnan

Yatan Pal Singh Balhara

Amitava Banerjee

Rahul Banerjee

Shweta Baral

Jenny Barrett

Fernando Barreyro

Alfred Bartolucci

Shantala Basawarajappa

Buddha Basnyat

Saurav Basu

Sharmishtha Basu

Jasbir Singh Bedi

Antoinette Bediako-Bowan

Ahmed Bedru

Geeta Behera

Gufran Beig

María Benzo-Iglesias

Jenny Berg

Natasa Berginc

Aneel Bhangu

Sujas Bhardwaj

Omesh Bharti

Sangeeta Bhatia

Anuj Kumar Bhatnagar

Romit Bhattacharya

Mouchumee Bhattacharyya

Nazim Bhimani

Zulfiqar Bhutta

Adriana Biney

Andreas L Birkenfeld

Per Bjorkman

James Blanchard

Matthieu Boisson

Dickens Borame

Josep Maria Borras

David Boulware

W Abdullah Brooks

Oscar Brousse

Clive Brown

Kyle Busse

Yanjia Cao

Paolo Cavoretto

Vineet Chadha

Joy Chakma

David Champredon

Tefu Chan

Palash Chandra Banik

Jie Chang

Ram Hari Chapagain

Pimlak Charoenkwan

Pragya Chaube

Sumita Chaudhry

Mu-Hong Chen

Vincent L Chen

Ka Wang Cheung

Saurabh Chhabra

Chi-Tsun Chiu

Jun-Hyung Cho

Young-Jae Cho

Yoon Hong Choi

Wenchi Chou

Ranadip Chowdhury

Parul Christian

Meijie Chu

Sheng-Chia Chung

David Churchill

Nicola Cirillo

Aleksandar Ćirović

Andrew Clark

Jamile Codogno

Andrew Copas

Chiara Corti

Nathalie Costedoat-Chalumeau

Andrea Cozzi

Javier Crespo

Ilaria Cuccu

Maryam Dadar

Omid Dadras

Shaonong Dang

Teguh Dartanto

Hemanshu Das

Aditya Dash

Michael David

Benjamin Davido

Claudio Dávila

Kevin M De Cock

Fiona De Londras

Avram Denburg

Sarang Deo

Richard J Derman

Kristen DeStigter

Saju Madavanakadu Devassy

Arnab Dey

Chetan Anil Dhamne

Federico Di Lello

Nadia Diamond-Smith

Haibo Ding

Huanjun Ding

Tao Ding

Merve Dizdar

Matthew Dodd

Thinley Dorji

John M Drake

Eduardo Duro

Susumu Eguchi

Linda S Ehrlich-Jones

Sarah Elitzur

Rachel Elliott

Zeynep Ergenc

Kang-Chih Fan

Li-Qun Fang

Panpan Fang

Eric Faragher

Mansoor Farahani

Pouya Farokhnezhad Afshar

Joveria Farooqi

Forough Farrokhyar

Rista Fauziningtyas

Gracia L T Fellmeth

Karla Fenton

Miranda M Fidler-Benaoudia

Florian Fischer

Tamara Fitzgerald

Alison Fogarty

Gian Forni

Palmira Fortunato dos Santos

J'Belle Foster

Delfin Lovelina Francis

Catherine Freeland

Anna Freni Sterrantino

Shin-ichiro Fujiwara

Tomoyasu Fukui

Albis Francesco Gabrielli

Rahul Gajbhiye

Mihir Gandhi

Vithiya Ganesan

Xu Gao

Samir Garg

Tushar Garg

Ian Gassiep

Amalia Gastaldelli

Nilesh Gawde

Nicholas Geard

Caspar Geenen

Marco Geraci

Tania Gergel

Elizabeth Gershoff

Jamal Gharekhani

Dirgha Jibi Ghimire

Jagat Jeevan Ghimire

Sushmita Ghimire

Kanjaksha Ghosh

Evangelos Giamarellos-Bourboulis

Andrea Giannini

Kevin Gibbs

Etienne Gignoux

Billie Giles-Corti

Surajit Giri

Jesudian Gnanaraj

Brian Godman

Daniel W Golden

Shoshanna Goldin

Gyanendra Gongal

Gerard Bryan Gonzales

José Luis Górriz

Shivaprasad Goudar

Shalini Govil

Anneke Grobler

Martin Grobusch

Jianmin Gu

Yong Gu

Tianjia Guan

Lusiele Guaraldo

Jia Guo

Lingyun Guo

Indrani Gupta

Pallav Gupta

Rajeev Gupta

Roop Gursahani

Tshewang Gyeltshen

Shams Shabab Haider

Claudia Hanson

Michael Harhay

Diane Harper

Sam Harper

Hamidreza Hasani

Lubna Hassan

Rohini Hawaladar

Lisa Hawke

Jian-Rong He

Yulei He

Oskari Heikinheimo

Jesper Heldrup

Helen Henderson

Christian Herzog

Saman Hewamana

Adam Hill

Galib Muhammad Shahriar Himel

Kenji Hirata

Wolfgang Hladik

Daniel J Hoffman

Eric P Hoffman

Susan Horton

Ahmed Hossain

Aniqa Tasnim Hossain

Sugeerappa Hoti

Tingting Hou

Zhiyuan Hou

Catherine Hoyt

Dongsheng Hu

Qiansheng Hu

Qing-Hai Hu

Sheng Huang

Yangmu Huang

Jörg W Huber

Peter Hudson

Syed Ali Husain

Adil Ali Hussein

George Imataka

Stephanie Irving

Md Rashedul Islam

Md Saiful Islam

Saule Issenova

Takanori Ito

Alane Izu

Kauser Jabeen

Bart Jacobs

Jagnoor Jagnoor

Tushar Jagzape

Murali Janakiram

Mohammad Javaherian

Benjarath Javed

Surangi N Jayakody

Prasanna Jayasekara

Ruwan D Jayasinghe

Asanka Jayawardane

Tarun Kumar Jeloka

Hannah Maria Jennings

Zhi-Cheng Jing

Tawanchai Jirapramukpitak

Denny John

Jacob John

Sangeeta Joshi

Sunil Kumar Joshi

Mikk Jürisson

Jessica Justman

Alamgir Kabir

Gilberto Kac

Nidhi Kadam

A B M Kamrul-Hasan

Peter Kamstra

Mona Kanaan

Rama Kandasamy

Suvi Kangas

Julie Kanter

Shuvra Kanti Dey

Sandip Kapur

Sujita Kar

Martha Kartasurya

Chollanot Kaset

Matt Kasman

Geetanjali Mahagundappa Katageri

Soundappan Kathirvel

Patricia Kavanagh

Anand Kawade

Alexander Kay

Eda Kaya

Ashish Kc

Mark Kelson

Zoe Kelson

Arthur Kemoli

Rachel M Kenney

Mohammad Kermansaravi

Amit Kumar Keshri

Suliman Khan

Tila Khan

Ali Khashan

Mohammad Khuroo

Tania King

Jens Klotsche

Hyla Louise Kluyts

Jennifer Marcelle Knight-Madden

Prakash Babu Kodali

Rajeev Zachariah Kompithra

Evangelos Kontopantelis

Benno Kreuels

Kaliannagounder Krishnamoorthy

Anand Krishnan

Suma Krishnasastry

Evangelos Kritsotakis

Stefan Kulnik

Alok Kumar

Ashwani Kumar

Suresh Kumar

Vivek K̀umar

Govindasamy Kumaramanickavel

Vinay Kumaran

Satyajit Kundu

Anura Kurpad

Myat Phone Kyaw

Sunee Lagampan

Ching Lung Lai

Yiwei Lai

Sham Lal

Prabhat Lamichhane

Zohra Lassi

Weidong Le

Ching-Chi Lee

Hak Seung Lee

Meihsuan Lee

Sander Lefere

Moisés León-Ruiz

Chi-kong Li

Dong Li

Qian Li

Zhihui Li

Jue Tao Lim

Kuang Hock Lim

Direk Limmathurotsakul

Chun-Yu Lin

Senlin Lin

Hanruo Liu

Xiaoxue Liu

Jara Llenas-García

Christopher A Loffredo

Watcharin Loilome

Anna Long

Christopher R Long

Qian Long

Daniel Lovell

Dorrain Low

Tiffany Lin Lucas

Claudio Luchini

Tom Lung

Aung K Lwin

Jianhua Ma

Lina Ma

Sally Mackay

Purnima Madhivanan

Amita Mahajan

Zaleha Mahdy

Tathagata Mahintamani

Zhiming Mai

Rosliza Manaf

Siddhartha Mandal

Adnan Mannan

Mohammad Ali Mansournia

Muchtaruddin Mansyur

Dale Mantey

Ravikanth Manyam

Xuhu Mao

Ivan Marbaniang

Paola Marchesini

Patrick Mark

Michael Marks

Glenn A Martínez

Alberto Mateo-Urdiales

Manu Mathur

Prashant Mathur

Ryan K McBain

Elizabeth McClure

Kenneth McElwain

Patrick McGann

Ryan McGrath

Mark McKinlay

Douglas McMillan

Barbara McPake

Raja Mehanna

Yatin Mehta

Shenghui Mei

Eelco F J Meijer

Jaideep Menon

Vikas Menon

Fiona Mensah

Sharayu Mhatre

Masoud Mirzaei

Aseem Mishra

Usha Misra

Sanchita Mitra

Mahima Mittal

Takao Miwa

Gazi Sakir Mohammad Pritom

Mohammad Javad Mohammadi

Shooka Mohammadi

Viswanathan Mohan

Sreelekshmy Mohandas

Prasanta Mohapatra

M Rubaiyat Hossain Mondal

Christopher J Morgan

Taeko Moriyasu

Ladda Mo-Suwan

Jon Moussally

Vamshi Muddu

Abdu Nafan Aisul Muhlis

Krishnendu Mukhopadhyay

Pradip Mukhopadhyay

Modest Mulenga

Anusheel Munshi

Nchangwi Syntia Munung

Takashi Murata

Khaled Musallam

Filip Mustač

Zia Ul Mustafa

Divya Nair

Manisha Nair

Pratibha Nair

Saritha Nair

Deipanjan Nandi

Cynthia C Nast

Sidrah Nausheen

Gathari Ndirangu

Nawi Ng

Eduardo Nilson

Tulika Nirmolia

Basile Njei

Bongani Brian Nkambule

Maurício Nobre

John Norrie

Bernard North

Kazunori Nozaki

Achmad Nurmandi

Jennifer Nyoni

Nicole Fortier O'Brien

Gilbert Sterling Octavius

Owen O'Donnell

Jongmin Oh

Sooyoung Oh

Kazunori Oishi

Gbemisola Oke

Kufre Joseph Okop

Roland Okoro

David Ong

Emanuel Orozco

Bryan Ortiz

Martijn Oudijk

Ramachandran Padmavati

Priyankar Pal

Ashish Kumar Pandey

Awadhesh Pandey

Basu Pandey

Deb Pandey

Elizabeth Davida Paratz

Catriona Parker

Zahra F Parker

Patrizio Pasqualetti

Anupam Khungar Pathni

Gopal Patidar

Muhammad Mainuddin Patwary

Atmaram Pandurang Pawar

Zhibin Peng

Henry Perry

Gianluca Perseghin

Olumuyiwa James Peter

Ben Omega Petrazzini

Tung Pham

Phuc Phan

Praphan Phanuphak

Vijay Pillai

Neethi P Pinto

Virginia Pitzer

Michael Plank

Laura Plata-Casas

Ramesh Poluru

Subramani Poongothai

Douglas Postels

Menno P Pradhan

Raja Pramanik

C Pramesh

Budi Prasetyo

Robin Prescott

Joan Puig-Barbera

Prasanta Purohit

Athina Pyrpasopoulou

Xiu Qiu

Rakesh Vadakkethil Radhakrishnan

Madiha Raees

Aminur Rahman

Atif Rahman

Mosiur M Rahman

Nurul Rahmawati

Priya Rajagopalan

Sudha Rajan

Subramania Raju Rajasulochana

Harsh Rajvanshi

Natalia Rakislova

Jaishree Raman

Alessandro Rambaldi

Kirtan Rana

Adam J N Raymakers

Mark Reger

Jürgen Rehm

Jianan Ren

Stefan Rennick-Egglestone

Henry Rice

Antonio Rivero-Juarez

Michael Stephen Robson

Helena Sofia Rodrigues

Alfonso J Rodriguez-Morales

Benjamin Rolland

Debendra Nath Roy

Anil Kumar Rudramuni Setty

Sanduk Ruit

Otto Ruokolainen

Gnana Sanga Mithra S

Ramesh S

Ramya S

Harshpal Sachdev

Parham Sadeghipour

Marc Saez

Senjuti Saha

Pragyan Monalisa Sahoo

Kirti Sundar Sahu

Nobuo Saito

Mayank Sakhuja

Ichiro Sakuma

Sarah Saleem

Claudio Salgado

Nathan Salvidge

Niharika Samala

Charles Sande

Leif Erik Sander

Daisuke Sano

Vishnu Prasad Sapkota

Sompob Saralamba

Patsharaporn Sarasombath

Swarup Sarkar

Sachin Chakradhar Sarode

Alyssa Sbarra

Elia Sechi

Yutaka Seino

Shahjada Selim

Y Selvamani

K L Seneviwickrama

Utpal Sengupta

Tulika Seth

Petra Sevcikova

Faraaz Shah

Mohsin Shah

Sharaf Ali Shah

Santhanam Sham

Deepthi N Shanbhag

Rajasubramaniam Shanmugam

Mohd Shannawaz

Amit Sharma

Nandini K Sharma

Rohit Sharma

Natasha Yasmin Sheikhan

Ji Shen

John A Shepherd

Md Monir Hossain Shimul

Nooshin Shirzad

Nirupama Shivakumar

Abu Bakkar Siddique

Gregory Simon

Amitabh Singh

Dhirendra Pratap Singh

Itu Singh

Pushpendra Pal Singh

Shweta Singh

Nidhi Singla

Yajai Sitthimongkol

Leon Snyman

Hamid Solgi

Biju Soman

Xingyue Song

Rufina Soomro

Sveinung Sørbye

Bundit Sornpaisarn

Priya Sreenivasan

Parikipandla Sridevi

Shiva Prakash Srinivasan

Shyam Srinivasan

Patumrat Sripan

Shyamkumar Sriram

Prateek Srivastav

Emily Chia-Yu Su

Rami Subhi

Alka Anand Subramanyam

Nikkil Sudharsanan

Kexin Sun

Tarun Kumar Suvvari

Gleb E Svinin

Sofia Ali Syed

Shahrad Taheri

Dar In Tai

Jackson Tan

Kelvin Bryan Tan

Julian Tang

Weiming Tang

Pranay Tanwar

Zachary Taylor

Yifokire Tefera

Chaitanya Tellapragada

Leanne Teoh

Rangaswamy Thara

Maile Thayer

Sreekanth Thekkumkara

Anish Thekkumkara Surendran

Sathish Thirunavukkarasu

Roger Thomas

Guy E Thwaites

Shiyam Sunder Tikmani

Beth Tippett-Barr

Dwi Hapsari Tjandrarini

Zheng Quan Toh

Siranda Torvaldsen

Toby Trahair

Zachary Tran

Dario Trapani

Paula M Trief

Thien Tan Tri Tai Truyen

I Jung Tsai

Muhammad Tufail

Taohsin Tung

Fahad Umer

Rahul Upadhyay

Haruki Usuda

Sakthivel Vaiyapuri

Pedro L Valenzuela

H Rogier van Doorn

Cornelis H van Werkhoven

Vidhya Venugopal

Ashish Verma

Rajesh Verma

Lakshmi Vijayakumar

Andrew Voetsch

Nguyen Lam Vuong

Marjan Walli-Attaei

Stephen Walsh

Dongqing Wang

Hongsheng Wang

Liang Wang

Mao-Shui Wang

Qiong Wang

Quan Wang

Xiaowen Wang

Yuke Wang

Zhenzhen Wang

Russell Ware

Marcia Weaver

Dannuo Wei

Lauren M Weinstock

Paul Weiss

Juan Wen

T Eoin West

Lana Whittaker

Kumudu Wijewardene

Mark Wilson

Esko Wiltshire

Vincent Wai Sun Wong

Fu-Zong Wu

Irene Wu

Nana Wu

Dong Xu

Yan Xuan

Uday Yadav

Juan Yang

Lianping Yang

George Yannis

Pengpeng Ye

Vijay V Yeldandi

Liv Yoon

Ritthideach Yorsaeng

Wei-Cheng You

Leanne Young

Hsiang-Yu Yuan

Zhangrui Zeng

Bo Zhang

Fengqing Zhang

Lele Zhang

Xinling Zhang

Mingye Zhao

Wei Zheng

Taiyang Zhong

Victor Zhong

Yuanpin Zhou

